# Who Participates in Seasonal Influenza Vaccination? Past Behavior Moderates the Prediction of Adherence

**DOI:** 10.4061/2011/148934

**Published:** 2011-08-23

**Authors:** Anna Ernsting, Sonia Lippke, Ralf Schwarzer, Michael Schneider

**Affiliations:** ^1^Department of Health Psychology, Freie Universität, 14195 Berlin, Germany; ^2^Jacobs Center on Lifelong Learning and Institutional Development, Jacobs University Bremen, 28759 Bremen, Germany; ^3^Warsaw School of Social Sciences and Humanities, 03815 Wroclaw, Poland; ^4^Boehringer Ingelheim Pharma GmbH & Co. KG, Occupational Health Services, 55216 Ingelheim, Germany

## Abstract

*Background.* Vaccination effectively prevents seasonal influenza. To promote vaccination adherence, it is necessary to understand the motivational process that underlies vaccination behavior. This was examined along with the moderating influence of past behavior on intention formation. *Methods.* German employees (N = 594) completed questionnaires at baseline and at 7-month followup. Regression analyses were conducted for mediation and moderated mediation. *Results.* Intention at Time 1 mediated the effect of risk perception, and positive and negative outcome expectancies on Time 2 vaccination. Past behavior moderated this effect: there was a mediation effect for risk perception and outcome expectancies only for those individuals who did not participate annually. *Conclusions. * Risk perception and outcome expectancies influenced intentions to receive vaccination, which in turn predicted participation. Hence, these social-cognitive variables could be targeted in vaccination campaigns to increase intentions. However, vaccination experience affected the formation of intentions and should be accounted for when developing interventions.

## 1. Introduction

Seasonal influenza is one of the most frequent contagious diseases worldwide. Every year the seasonal flu can lead to suffering, illness, or death. Moreover, it causes major societal (e.g., consultations, hospitalization, and deaths) and economic (e.g., absenteeism) problems [1–3]. Annual influenza vaccination is considered the most effective way to prevent the onset of influenza and its complications, and it is officially recommended by the World Health Organization [4] and national institutions [5, 6] amongst others for older adults and individuals working in crowded settings.

Despite this recommendation, participation rates in Germany [3] as well as in the USA [6, 7] are lower than desired and should be increased. A profound knowledge of the mechanism involved in the target behavior is the basis for the development of effective preventive programs [8, 9]. Thus, it is worthwhile to investigate (a) social-cognitive factors that may influence vaccination motivation and participation, and (b) how intention formation can also be affected by past behavior [10]. This would be consequential for the design of preventive programs. Hence, the present study pursued these research questions in light of social-cognitive theories of health behavior [11, 12].

Theories of health behavior change focus on the prediction and modification of the adoption and maintenance of health behaviors [13]. *Risk perception* and outcome expectancies are considered to be major motivational predictors of behavioral intentions [14–16] and are part of various theories on health behavior. However, in the context of vaccination, variables closely related to the risk construct, conveying a strong affective component, turned out to be better predictors than mere “thoughts” [14, 17]. Worry about influenza is such a construct and, therefore, serves as an indicator for perceived risk. *Outcome expectancies* represent the expected consequences of an action and are part of the social cognitive theory (SCT) [18] and the health action process approach (HAPA) [12, 19]. A distinction is made between *positive outcome expectancies* (“If I get a flu shot, then I will have the best protection against the flu”) and *negative outcome expectancies* (“If I get a flu shot, then I will suffer from side effects”). Positive outcome expectancies promote, whereas negative outcome expectancies inhibit an intention formation [20–22]. Hence, a decisional imbalance in favor of positive outcome expectancies helps to form an intention. In turn, an *intention* represents a significant predictor of the target behavior [23–25]. Therefore, it was hypothesized that *risk perception and positive outcome expectancies are positively associated with intention, and negative outcome expectancies are negatively correlated with intention. Intention, in turn, is supposed to mediate between the motivational predictors (risk perception and outcome expectancies) and subsequent behavior (participating in the vaccination) (hypothesis 1).*

*Past behavior* is usually closely associated with subsequent behavior and has been found to be the best predictor of later adherence [26–28]. Beyond that, findings indicated its influence on cognitive processes concerning the initiation, execution, or control of behavior [10, 28] which is addressed in the current study. If a behavior is carried out *frequently* in a *stable context*, cognitive processes can be bypassed, and responses are performed rather automatically. Responses are carried out quickly and require no conscious decision making and thinking whilst remaining goal directed and functional. With repetition, behavior is increasingly under control of situational cues, which then are sufficient to trigger an automatic process. In contrast, a new or infrequent behavior—especially in unstable settings—requires controlled, deliberate processing as individuals are assumed to review their beliefs before acting [10, 29].

Obtaining a flu shot represents an infrequent behavior (once a year). Nevertheless, given a repetitive performance in a stable context, it is assumed that the cognitive process of intention formation can also adopt an automatic nature. If a short message about influenza vaccination (= situational cue) is presented to people who went for a flu shot annually over the last several years (= repetitive behavior) by the workplace health service (= stable setting), the motivation to get vaccinated should be almost automatic [10, 29]. In contrast, people who participated only infrequently or not at all in the past should contemplate on their personal risk and the pros and cons of a vaccination before they form a behavioral intention. Thus, it is hypothesized that *past behavior moderates the indirect effect of positive outcome expectancies, negative outcome expectancies, and risk perception on later behavior via intention. The less an individual has participated in the past, the higher the impact of the motivational variables on intention formation (hypothesis 2).*

## 2. Method

### 2.1. Sample and Procedure

Participants of the longitudinal questionnaire study were individually approached and recruited in a large German company. Data at *Time *1 (*T*1) were collected (a) before the vaccination campaign started for four days in front of the cafeteria and (b) whilst the vaccination campaign was running, but *before people got the flu shot* in the occupational health service (September 2009). Completion of the form took about 10 minutes. A note on the questionnaire informed participants where to look for information about the vaccination campaign on the intranet. Vaccination was administered by the occupational health service the same way as every year. The company's work committee approved the study for data privacy and ethical standards, and it was conducted in line with the ethical guidelines of the German psychological society.

*N* = 1,466 employees participated at *T*1 (out of 11,434 employees in this company), response rate 12.8%), 521 (35.5%) participants were women and 810 (55.3%), men (135/9.2% not specified). Mean age was 41.64 years (SD = 9.77), and age ranged from 16 to 67 years. 

The follow-up questionnaire at *Time *2 (*T*2) was distributed seven months later via internal mail, when the influenza season was over (April 2010). Only those were contacted who had given consent at Time 1. Out of 1,214 potential participants, 594 employees responded (dropout rate 53.7%): 55% were men, 45% were women. Mean age was 43.1 years (SD = 9.1), and age ranged from 16 to 61 years of age. The original sample at *T*1 (*N* = 1,466) differed from the longitudinal sample (*N* = 594) in self-reported past behavior (*T*1 sample: *M* = 3.4; *T*2 sample: *M* = 3.6; *t* = −2.32; *P* = .02). Individuals who had obtained seasonal flu vaccination more frequently in the past were more likely to remain in the study. No significant differences were found regarding all other variables (see following paragraph). 

### 2.2. Variables and Measures

At *T*1, positive and negative outcome expectancies, risk perception, intention, and past behavior were assessed. Vaccination behavior was assessed at *T*2. The implemented scales were adapted from validated scales [14, 30, 31], partly developed by seasonal influenza experts at the Robert Koch Institute (RKI) [32]. Responses were given on 4-point Likert scales, from 1 (*strongly disagree*) to 4 (*strongly agree*), if not reported differently in the following.

*Risk perception* was measured with the item “I am worried that I will get the flu this year” [14].

*Positive outcome expectancies* were assessed with six items (Cronbach's *α* = .58). Two items were adapted from Schwarzer et al. [31], for example, “If I get a flu shot this season, then I promote my health.” Four items were adapted from RKI [32]. Participants had to comment on them, for example, “Influenza vaccination decreases the risk of catching the flu.”

*Negative outcome expectancies* were measured with two items [32], for example, “I won't get a flu shot because I'm afraid of side effects” (*r*² = .63).

Intention was measured with the item [30] “I intend to get a flu shot this season.” Responses were given on a 7-point Likert scale, from 1 (*strongly intend to*) to 7 (*do not intend at all*). 

*Self-reported past behavior *[32] at *T*1 was assessed with the item “How often did you get the flu shot within the last 5 years?” Responses were given on a 5-point scale: 1 (*not at all*), 2 (*once*), 3 (*twice*), 4 (*more than twice, but not annually*), and 5 (*annually*). 

*Self-reported behavior *at *T*2 was assessed with the item “Did you get a flu shot during the last flu season (within the last six months)?” Response categories were 1 (*yes*) and 2 (*no*). In Table 1, means, standard deviations, and correlations are presented. 

### 2.3. Analyses

Regression analyses were performed with SPSS (version 18.0) to examine mediation and moderated mediation, using standardized scores [33]. A mediation analysis was conducted to address *hypothesis 1,* and a moderated mediation analysis to address *hypothesis 2* [34, 35]. Mediation analyses were chosen to investigate how and why an effect occurred, that is, receiving a flu shot [34]. A *mediation effect* is expressed in an *indirect effect*. An independent variable affects a dependent variable via a third variable (mediator). The strength or the form of a mediation effect may be moderated by a third variable (moderator variable) [34]. This is called *moderated mediation* and can be expressed as a *conditional indirect effect. *Preacher et al. [36] specified, among others, a model that describes the influence of a moderator on the relationship between the independent variable and the mediator. The particular value of the moderator, at which the mediation effect is conditional at a set level (*α* = .05), can be identified with the Johnson-Neyman technique [36].

SPSS macros by Preacher and Hayes [35] were used to analyze the indirect effect (hypothesis 1) and a conditional indirect effect (hypothesis 2). Conditional indirect effects were expressed in interaction terms, for example, past behavior × risk perception. Regression analyses for mediation and moderated mediation consisted of two regression analyses that were conducted in succession: first the mediator model and then the dependent variable model. Analyses for moderated mediation were conducted separately for each predictor, in each case controlling for the other predictors. The effect size of the logistic regression was reported with Nagelkerke *R* ^2^. Because less than 5% of values were missing, no missing value imputation was performed [37].

## 3. Results

### 3.1. There Is an Indirect Effect of Risk Perception, Positive and Negative Outcome Expectancies at *T*1 on Behavior *T*2 via Intention *T*1 (Hypothesis 1)

**Figure 1:**
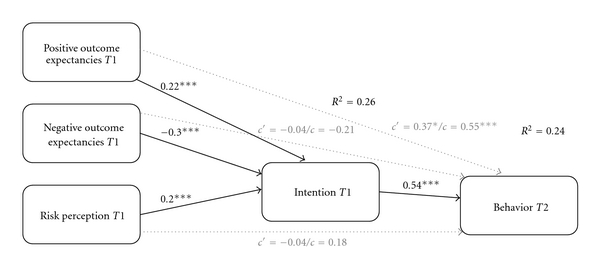
Mediation analysis (*N* = 552). Indirect effect of risk perception *T*1, negative outcome expectancies *T*1 on behavior *T*2 via intention *T*1. Partial mediation effect for positive outcome expectancies *T*1. Controlled for past behavior *T*1. Note: ***P < .001; *P < .05; *c'* = direct effect of independent variable on dependent variable; *c* = total effect of independent variable on dependent variable.

Results of the mediation analysis (Figure 1) demonstrated that risk perception (*β* = .20) and positive (*β* = .22) and negative (*β* = −.30) outcome expectancies were strongly associated with intention at *T*1 (*P* < .001). In turn, intention *T*1 predicted behavior at *T*2 (*β* = .54; *P* < .001). Intention at *T*1 mediated completely the influence of risk perception (*c'* = −.04; *P* = .85) and negative outcome expectancies (*c'* = −.04; *P* = .82) on behavior at *T*2. There was a partial mediation effect for positive outcome expectancies (*c'* = .37; *P* = .03). These findings provided support for the first hypothesis. Past behavior was included as a covariate, but it had no predictive value (*β* = .29; *P* = .11).

### 3.2. Moderated Mediation Analyses (Hypothesis 2)

**Figure 2:**
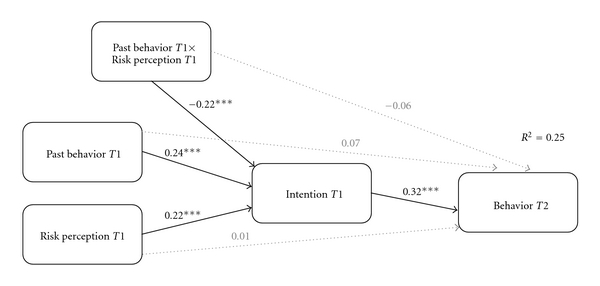
Moderated mediation analysis (*N* = 552). Past behavior *T*1 moderates the indirect effect of risk perception *T*1 via intention *T*1 on behavior *T*2. Note: ***P < .001.

However, the influence of past behavior on later participation was rather seen in its function to moderate the process of intention formation (hypothesis 2). Results of moderated mediation analysis for *risk perception* supported the assumption of a *conditional indirect effect* (*R* ^2^ = .25). Past behavior *T*1 moderated the mediation effect, which is displayed in Figure 2. 

**Figure 3:**
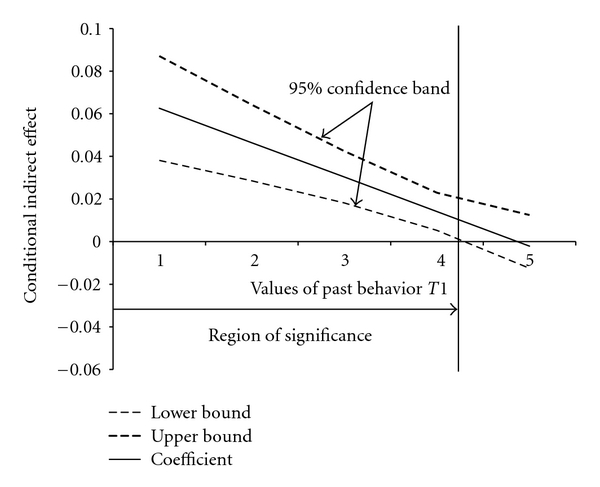
Moderated mediation analysis with Johnson-Neyman technique (risk perception). Note: the horizontal line indicates the lack of an indirect effect (no mediation). The vertical line represents the boundary of the region of significance (4.3).

The Johnson-Neyman analysis revealed that there was only an indirect effect of risk perception *T*1 on behavior *T*2 for people who scored lower than 4.3 on the scale for past behavior (*P* = .05): a mediation effect was only indicated if an individual had “not at all” (1), “once” (2), “twice” (3), and “more than twice, but not annually” (4) received a flu shot within the last five years. There was no indirect effect if someone was vaccinated “annually within the last 5 years” (5).  Figure 3 illustrates the conditional indirect effect at all values of the moderator with a 95% confidence band.

For *negative outcome expectancies*, the same result pattern (negative outcome expectancies × past behavior: *β* = .16; *P* < .001; past behavior at *T*1 = 4.3; *P* = .05) was found. 

The analyses for *positive outcome expectancies* also indicated a conditional indirect effect (positive outcome expectancies × past behavior: *β* = .24; *P* < .001). However, results differed in that no indirect effect was indicated if an individual scored higher than 4 on the scale of past behavior (*P* = .05), that is, an individual had received a flu shot “more than twice, but not annually” (4) and “annually within the last 5 years” (5).

## 4. Conclusion

The first aim of the study was to identify the social-cognitive processes that determine vaccination behavior. Findings supported the first hypothesis: the higher the risk perception of seasonal influenza is, the more positive outcomes—respectively, the fewer negative outcomes—in conjunction with obtaining a vaccination were reported. As a result of these associations, the vaccination motivation becomes higher, and later participation becomes more likely. The *complete mediation effect* for *risk perception* and *negative outcome expectancies* confirmed their limited influence on vaccination behavior via the formation of intentions. Risk perception and negative outcome expectancies can be seen as rather distal antecedents of intention and might set the stage for a more sophisticated reflection of potential action [12]. Hence, the influence of risk perception and outcome expectancies on health behavior is only indirect. In contrast, the *partial mediation effect* for *positive outcome expectancies* revealed that perceived positive consequences of getting a vaccination were of motivational importance but also had a direct effect on behavior performance [18]. Overall intention represented a good predictor for later participation. This leads to the conclusion that interventions targeting risk perception and outcome expectancies may effectively enhance vaccination motivation and subsequent participation. This could be done by providing information about the risk and potential severity of the infection (risk perception). Outcome expectancies could be targeted by discussing their options—no vaccination, preventive, and curative methods—with the respective consequences, for example, data on safety, effectiveness, and putative side effects of the vaccine [38].

However, moderated mediation analyses (hypothesis 2) revealed that past behavior presented a substantial moderator in this interplay; intention formation based on perceived risk of influenza, perceived benefits, and costs of vaccination depended on past vaccination behavior. All those who went to the influenza inoculation annually (and regarding positive outcome expectancies of those stating “more than twice, but not annually”) did not base their decision on the social-cognitive variables mentioned before. This may lead to the conclusion that for those people, intentions were formed rather automatically. Vaccination motivation appeared to be more under control of environmental stimuli, that is, the situational cue (= note on the questionnaire) that was presented in a stable setting (= occupational health service). This interpretation would be in line with the findings by Ouellette and Wood [10], revealing that given a stable setting, a relatively infrequent type of health behavior can also develop habitual tendencies. This should be encouraging for all practitioners. But whether vaccination can actually (and correctly) be labeled as a habit needs to be further investigated with adequate measurement [29]. In sum, this leads to the conclusion that future interventions should attend to varying needs in order to operate best. However, these suggestions need to be tested in future experimental intervention studies. When doing so, it is recommended to account for the moderating effect of past behavior. 

Study results must be considered in light of potential limitations. Vaccination behavior was measured by anonymous self-report that may impair validity. Objective measures, for example, medical reports of vaccination, may be preferable but were not available. However, studies on other health behaviors demonstrated validity of self-report measures [39]. Furthermore, risk perception was assessed with a single-item scale. This was done for economic and theoretical reasons; it can be assumed that a content-valid item can assess a narrow target construct just as well as a multiitem scale, in particular as vaccination adherence represents a highly specific behavior [14, 40]. Next, there was a systematic dropout of participants at *T*2 which may be due to (a) the different recruitment strategies at Time 1 (face-to-face) and Time 2 (internal mail), presumably leading to lower commitment of participants to the study at Time 2 and (b) the time of Time 2 assessment, as there were Easter holidays and many employees were unavailable. Nonetheless, a similar moderated mediation effect would be expected if those individuals would have remained in the longitudinal sample. As always, the present findings are limited to the study context and require replication before they can be generalized. 

In conclusion, the current large and longitudinal study may lead to a better understanding of vaccination behavior, especially by pointing out the influence of an individual's vaccination biography. The findings should be considered when future vaccination campaigns are developed and evaluated.

## Figures and Tables

**Table 1:** Risk perception, positive/negative outcome expectancies, past behavior, intention *T*1, and behavior *T*2 (means, standard deviations).

Variable	Risk perception *T*1	Negative outcome expectancies *T*1	Positive outcome expectancies *T*1	Past behavior *T*1	Intention *T*1	Behavior *T*2	*M*	SD
Risk perception *T*1^a^							2.4	.83
Negative outcome expectancies *T*1^a^	.05						1.2	.05
Positive outcome expectancies *T*1^a^	.12*	−.28**					3.2	.04
Past behavior *T*1^a^	−.03	−.19**	.17**				3.5	1.4
Intention *T*1^a^	.18**	−.34**	.33**	.31**			5.5	1.1
Behavior *T*2^a^	.06	−.28**	.26**	.20**	.40**			

Note: *N* = 594. *P < .05. **P < .01. ^*ª*^Range fully exhausted.
